# 75 Years of the Ravitch Procedure: A Historical Report and Review of Outcomes

**DOI:** 10.1016/j.atssr.2025.03.020

**Published:** 2025-04-16

**Authors:** Dianela Perdomo, Albert Leng, Deven Patel, Stephen C. Yang, Jinny S. Ha

**Affiliations:** 1 Division of Thoracic Surgery, Department of Surgery, Johns Hopkins School of Medicine, Baltimore, Maryland

## Abstract

**Background:**

In 1949, Dr Mark Ravitch described an open surgical approach for correcting pectus excavatum with bilateral excision of the deformed costal cartilages followed by a transverse wedge osteotomy. In honor of the procedure’s 75th anniversary, this report reviews its development and patient outcomes at the Johns Hopkins Hospital (Baltimore, MD).

**Methods:**

The Chesney Medical Archives and Epic databases were reviewed to identify patients who underwent surgical repair for pectus excavatum from 1947 to 2024. Patient and procedure characteristics were recorded. The Mark Ravitch and Alex Haller collections, which included personal notes and recordings of the procedure, were reviewed.

**Results:**

From the Johns Hopkins Hospital surgical logbooks (1947-1971), 217 open repair (Ravitch) operations were performed for pectus excavatum. The mean patient age was 7.0 (SD 6.8) years (range, 3 months to 42 years). 72.5% (n = 158) of patients were male, and 2.3% (n = 5) of cases were redo operations. From 2016 to 2024, 35 Ravitch and 271 Nuss repairs were performed. Patients who underwent Ravitch procedures were older (mean age, 36.0 [11.9]] years vs 15.8 [2.2] years; *P* < .0001) and included more redo operations (45.7% vs 1.5%; *P* < .0001). Nuss repairs were shorter in mean length of stay (2.4 [1.2] days vs 4.3 [2.0] days; *P* < .0001).

**Conclusions:**

The Ravitch procedure is generally performed in adult patients rather than in the younger pediatric patients who underwent surgery at its introduction in 1949. This procedure is typically reserved for patients in whom conservative management with a Nuss procedure has failed. The Ravitch procedure has endured as a safe surgical approach with favorable outcomes for the surgical management of complex chest wall deformities.

**In Short**

▪ Mark Ravitch revolutionized pectus excavatum surgery with his 1949 technique, which remained the gold standard for decades.
▪ The minimally invasive Nuss procedure has since largely replaced the Ravitch approach.
▪ Although Ravitch performed the operation on pediatric patients and advocated for intervention early in development, his procedure is now generally reserved for adults, complex cases, or patients in whom Nuss procedures failed.

In 1949, Mark Ravitch published his first case series on the surgical correction of pectus excavatum.^1^ The operation has since been known as the Ravitch procedure and is performed most often by cardiothoracic surgeons at our institution (Johns Hopkins Hospital, Baltimore, MD). In recognition of the 75th anniversary of its introduction and Dr Ravitch’s contributions to the field of surgery, this report describes the origins of the operation at the Johns Hopkins Hospital and its enduring impact.

## Methods

The Mark Ravitch and Alex Haller collections were reviewed. Patients who underwent surgical treatment of pectus excavatum were identified from the Alan Mason Chesney Medical Archives (1947-1976) and Epic databases (2016-2024). Demographic characteristics and surgical outcomes were analyzed. Patients who underwent the Ravitch repair were compared with patients who underwent the Nuss repair. Student *t* tests and χ^2^ tests were used to compare continuous and categorical variables, respectively; *P* ≤.05 was considered significant. Institutional Review Board approval was obtained (IRB00419752).

## Results

### Mark Ravitch: Early Life and Training

**Figure 1:**
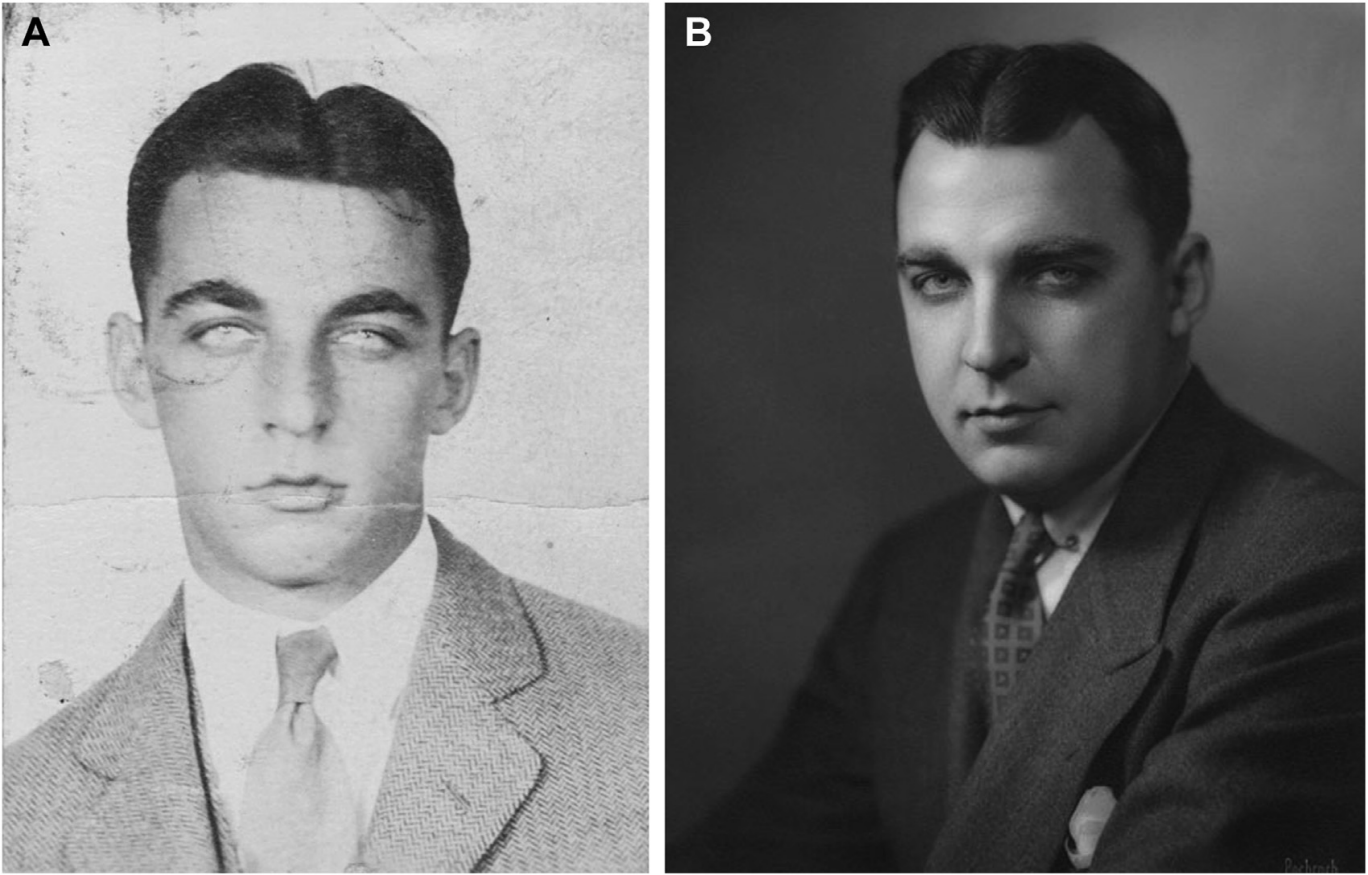
(A) Marcus M. Ravitch, student record portrait (1930). (B) Marcus M. Ravitch, portrait (1936). Courtesy of the Chesney Archives of Johns Hopkins Medicine, Nursing, and Public Health.

Marcus M. Ravitch was born on September 12, 1910, in New York and was the son of Russian immigrants. He developed an early interest in medicine, influenced by a number of physicians on his mother’s side, and earned his BA from the University of Oklahoma in 1930, where his uncle was a mathematics professor.^2^ In 1930, he matriculated at the Johns Hopkins School of Medicine and in 1934 began a general surgery internship under Dean Lewis, the second Chairman of the Department of Surgery, who succeeded William Halsted (Figure 1).^3^

After completing his residency in 1943, Ravitch was commissioned as a major in the Army during World War II. He directed the staff of surgeons of the 56th General Hospital and was present during the Battle of the Bulge, the last German offensive campaign on the western front. American troops had an estimated 75,000 casualties, and in service of his countrymen, Ravitch was reported to have operated continuously for 72 hours.^4^

**Figure 2:**
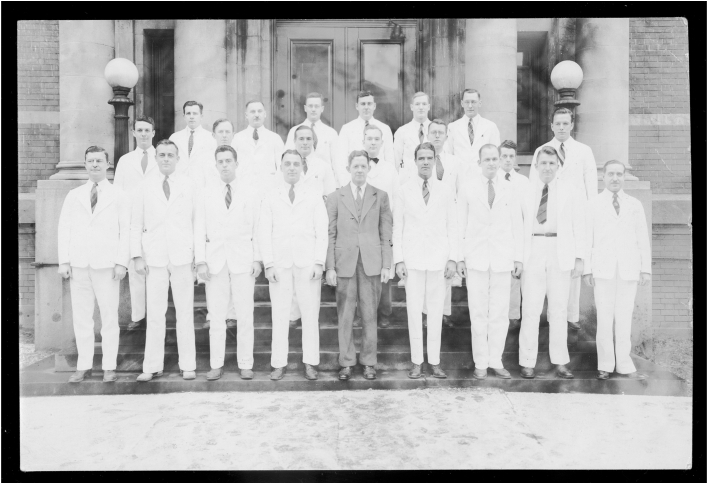
House staff portrait, 1946. Dr Alfred Blalock is in the center of the first row, and Dr Ravitch is to his right. Courtesy of the Chesney Archives of Johns Hopkins Medicine, Nursing, and Public Health.

In 1946, he returned to Baltimore as the first director of the Johns Hopkins blood bank under Alfred Blalock (Figure 2). He was drawn primarily to pediatric surgery, an interest that began when he was a medical student and that is credited to his first surgical mentor, Monty Farrar, who taught an elective course on pediatric surgery.^3^ Ravitch’s interest in defects of the chest wall is credited to another mentor, Edwards A. Park, whom he met during his second intern year in pediatrics. After a surgical internship, Ravitch applied to pediatrics because he believed that he did not measure up to his competition at a time when surgical training followed the pyramidal system. His peers had fathers who were prominent surgeons, of which he later said, “Those things weighed heavily…and I thought it would be suicidal to enter into competition with them on an equal basis…”^3^ After returning to Johns Hopkins, he was encouraged by Park to study chest wall deformities, in view of the limited understanding of the subject at the time.^3^

### Early Contributions to the Treatment of Pectus Excavatum

Although repair of pectus excavatum is closely associated with Dr Ravitch, he was not the first to address the condition surgically. In 1911, Meyer^5^ introduced the first technique, which involved resection of the second and third costal cartilages to relieve persistent dyspnea in a 16-year-old boy, although the patient’s symptoms persisted postoperatively. Several modifications followed, including sternotomies and the use of external sternal traction.^6^ Ochsner and DeBakey^6^ published a comprehensive review of the literature in 1939 that advocated for sternal mobilization with external traction given the absence of reported deaths, compared with a cumulative operative mortality of 20% to 25% for cartilage resection with or without sternotomy.

Ravitch’s approach differed primarily from those of his peers in that he advocated for resection of all of the malformed costal cartilages to prevent progression and recurrence of the excavation.^1^ He also advocated for early intervention during the first years of life to increase the patient’s chances of attaining a normal appearance as the thoracic cavity continued to develop.^1^ After resection of the cartilages, the xiphisternal junction was transected, and a wedge osteotomy was made at the manubriosternal junction. The sternum rested freely except for its attachment to the manubrium with silk sutures, and the ends of the resected cartilages were left free. No external traction was used, although this was used in 2 patients and abandoned after the second patient died of a pyogenic infection.^1^

### Analysis of Archival Patient Records (1947-1976)

**Figure 3:**
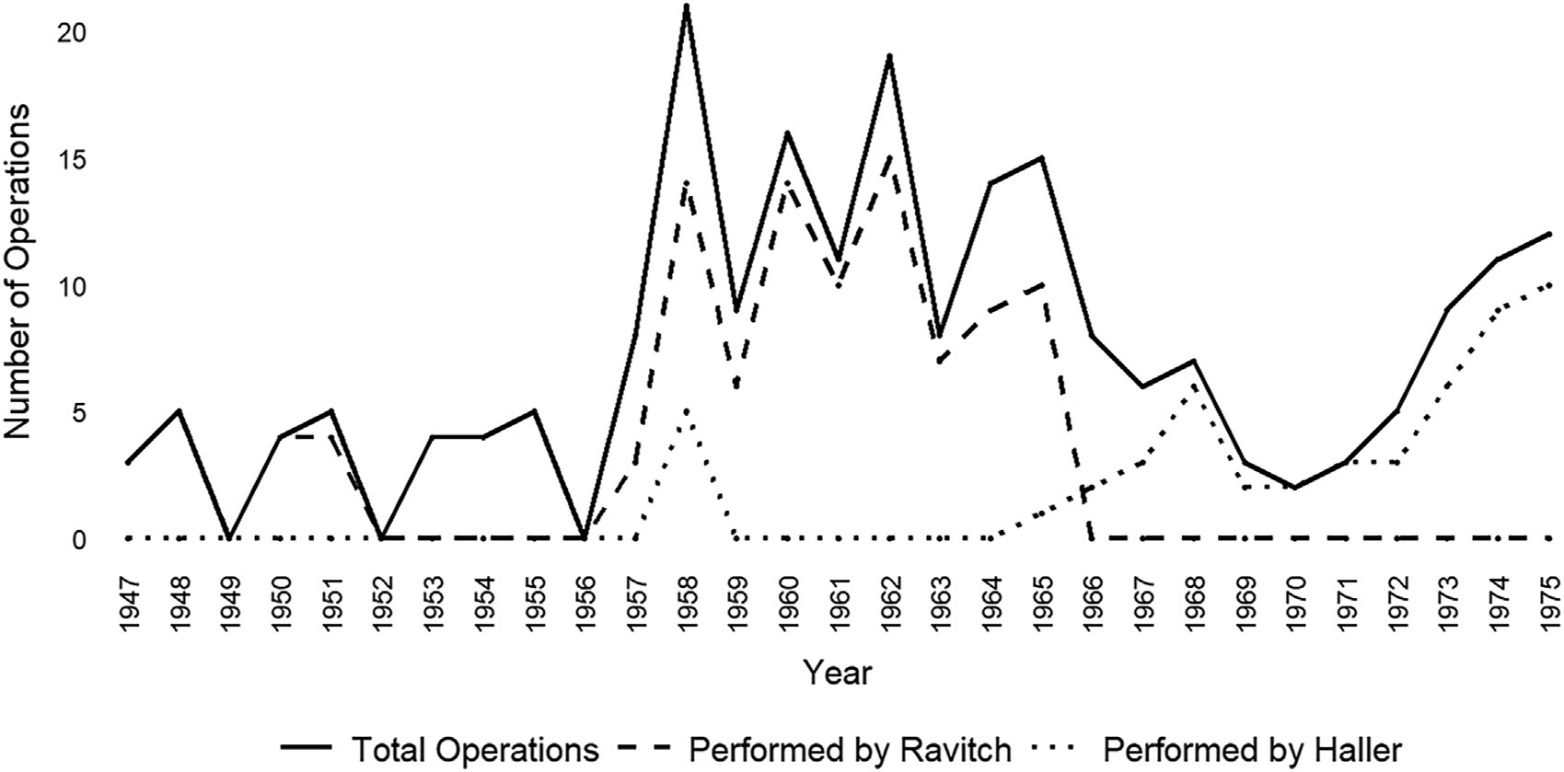
Ravitch repairs for pectus excavatum at the Johns Hopkins Hospital, Baltimore, Maryland (1947-1976). The increase in operations in the 1950s was driven by Ravitch’s return to Johns Hopkins in 1956 after a professorship at Columbia University (New York, NY), and the decrease in 1966 was driven by his departure to the University of Chicago (Chicago, IL) to serve as the Division Chief of Pediatric Surgery.^3^

A total of 217 Ravitch procedures for pectus excavatum were performed from 1947 to 1976 (Figure 3).^3^ The average patient age was 7.0 (SD 6.8) years (n = 166; range, 3 months to 42 years), and 72.5% (n = 158) of the patients were male. Male patients were significantly younger than female patients (average age: male patients, 6.3 years; female patients, 9.0 years; *P* = .043). For cases with racial demographic data (n = 126), 123 (97.6%) patients were White, 2 were African American, and 1 was Asian. A total of 2.3% of surgical procedures were redo operations. The average time to reoperation was 4.3 years.

Ravitch performed 49.3% (n = 107) of surgical procedures during this period. In 200 cases, the primary surgeon’s area of specialization was established through available biographic data. Most of these operations (90.0%; n = 180) were performed by individuals specializing in pediatric surgery, followed by surgeons specializing in cardiothoracic surgery (9.5%; n = 19). No patients required operative intervention for management of a complication. Individual records were inaccessible for the comprehensive series, and therefore assessment of operative variables was not possible. One case of fatal empyema was reported in the first series of 7 patients, and the average length of stay was 9 days.

After Ravitch left for the University of Chicago (Chicago, IL) in 1966, the procedure was performed primarily by his mentee, Alex Haller, who also made significant contributions to pectus excavatum surgery through the development of the Haller index, which provided an objective measure to assess the severity of the excavation.^7^

### Analysis of Electronic Medical Record Cases (2016-2024)

**Figure 4:**
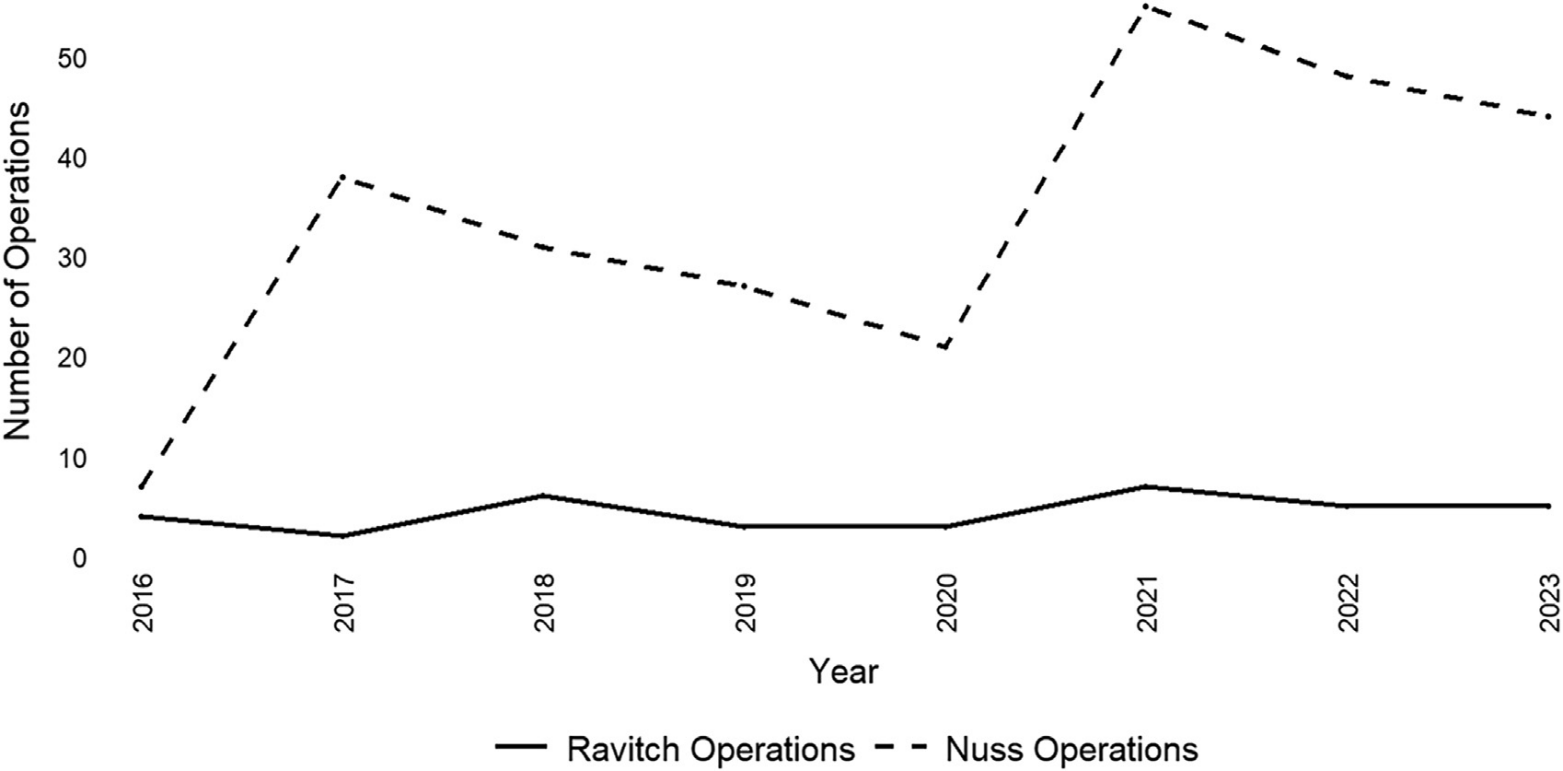
Ravitch and Nuss operations performed for pectus excavatum at the Johns Hopkins Hospital in Baltimore, Maryland (2016-2024).

From 2016 to 2024, 35 Ravitch procedures were performed (Figure 4). The average patient age was 36.0 (11.9) years (n = 35; range, 23-77 years). The average Haller index was 4.5 (1.3) for 28 cases with available imaging; 60.0% (n = 21) of the patients were male. There was no significant difference in the average age of male and female patients at the time of surgery (male patients, 37.8 [14.3] years; female patients, 33.3 [5.2] years; *P* = .27). The average length of stay was 4.3 (2.0) days. Most patients were White (88.6%; n = 31), followed by 5.7% (n = 2) who identified as Black. A total of 16 (45.7%) patients had redo operations: 5 patients had previous Ravitch repairs, 2 had previous modified Ravitch repairs, 4 had previous Nuss repairs, 4 had both Ravitch and Nuss repairs, and 1 underwent correction with a silicone implant. All operations were performed by a thoracic surgeon. Patients who underwent Ravitch repair from 2016 to 2024 were significantly older than patients who underwent repair from 1947 to 1976 (1947-1976, 7.0 [6.8] years; 2016-2024, 36.0 [11.9] years; *P* < .0001).

From 2016 to 2024, 271 Nuss repairs were performed (Figure 4). The average patient age was 15.8 (2.2) years (n = 271; range, 11-34 years). The average Haller index was 4.7 (1.7) for 267 patients with available imaging; 86.0% (n = 233) of the patients were male. There was no significant difference in the average age of male and female patients at the time of surgery (male patients: 15.9 [2.2] years; female patients, 15.4 [2.5] years; *P* = .21). The average length of stay was 2.4 (1.2) days. Most patients were White (90.0%; n = 245), followed by 4.0% (n = 10) who identified as Asian, 3.0% (n = 8) as “other,” and 1.0% (n = 4) as Black. A total of 1.5% of cases were redo operations. All surgical procedures were performed by a pediatric surgeon.

Patients who underwent Ravitch procedures were significantly older than patients who underwent Nuss procedures (average age: Ravitch, 36.0 [11.9] years; Nuss, 15.8 [2.2] years; *P* < .0001) and had a longer length of stay (Ravitch, 4.3 [2.0] days; Nuss, 2.4 [1.2] days; *P* < .0001). There was no difference in the Haller index between the groups (Ravitch, 4.5 [1.3]; Nuss, 4.7 [1.7]; *P* = .55). Among cases with racial demographics (1947-1976, n = 126; 2016-2024, n = 306), the percentage of White patients undergoing surgery was significantly different (1947-1976, 97.6%; 2016-2024, 90.2%; *P* = .008).

## Comment

The surgical management of pectus excavatum has evolved significantly from its conservative origins to the development of various operative interventions. When Mark Ravitch introduced his surgical approach in 1949, he emphasized complete cartilage resection and sternal mobilization, with no additional wiring or stabilization apart from silk sutures approximating the wedge osteotomy.^1^ Previous operations ranged from partial cartilage resections and sternal osteotomies to the use of external traction wiring that achieved limited success and were often associated with severe complications.^6^ Ravitch reported significantly lower rates of mortality and recurrence, thus rendering his approach the gold standard in pectus excavatum surgery throughout most of the 20th century.

Although recurrence was rare, cases requiring reintervention were complicated by severe scarring and calcification in areas where cartilage was removed. Furthermore, Ravitch’s advocacy for early intervention was associated in some patients with the development of acquired thoracic dystrophy, a debilitating restrictive lung disease caused by damage to the rib growth plates. Despite Ravitch’s many contributions, this represented a significant failure of the procedure. In 1998, Donald Nuss and colleagues^8^ introduced the minimally invasive Nuss procedure, which required no cartilage resection or osteotomy but relied on a substernal bar insertion that was flipped to elevate the sternum. The procedure gained favorability for the treatment of pectus excavatum given similar rates of symptomatic and cosmetic satisfaction with a shorter length of stay. Although current practice includes thoracoscopic bar insertion, early iterations involved blind placement, which resulted in the death of a patient who incurred ventricular injury. A modified Ravitch approach was also introduced and aimed to preserve more of the native cartilage and soft tissue.^9^ Although no rigorous comparison was made between the modified Ravitch approach and the Nuss repair, the latter was likely embraced more readily given its perception as being less invasive. At Johns Hopkins, the Ravitch procedure has become reserved for patients in whom Nuss procedures have failed or adults who are no longer Nuss procedure candidates.

The indications for undergoing surgery today encompass symptomatic cases and those with cosmetic dissatisfaction. Patients who underwent Ravitch repair from 1947 to 1971 were significantly younger and often underwent surgery in the first years of life, as Ravitch advocated. Although in terms of demographics, patients undergoing this repair have historically been predominantly White, the recent series (2016-2024) includes a greater percentage of patients from racial minorities. Future work is needed to investigate whether this change reflects the epidemiology of the condition or whether there is disparate access to surgical care in patients from racial minority backgrounds.

On the 75th anniversary of the Ravitch procedure, we celebrate a surgeon whose contributions reshaped chest wall surgery. Dr Ravitch developed a technique that emphasized safety and improved patient outcomes, thereby serving as a foundation for future advancements in the field.^1^ He is also recognized for his pioneering influence in pediatric gastrointestinal surgery, the introduction of the surgical stapler, and his work as a surgical historian.^3^^,^^10^ On his death resulting from prostate and colon cancer in 1988, his remarkable collection of medical texts was donated to the Falk Library in Pittsburgh, Pennsylvania.^10^ Ravitch’s legacy endures in the generations of surgeons he trained and in the many others he continues to inspire.
